# Transcriptional Regulation of the Creatine Utilization Genes of *Corynebacterium glutamicum* ATCC 14067 by AmtR, a Central Nitrogen Regulator

**DOI:** 10.3389/fbioe.2022.816628

**Published:** 2022-02-09

**Authors:** Hao Zhang, Zhilin Ouyang, Nannan Zhao, Shuangyan Han, Suiping Zheng

**Affiliations:** ^1^ Guangdong Key Laboratory of Fermentation and Enzyme Engineering, School of Biology and Biological Engineering, South China University of Technology, Guangzhou, China; ^2^ Guangdong Research Center of Industrial Enzyme and Green Manufacturing Technology, School of Biology and Biological Engineering, South China University of Technology, Guangzhou, China

**Keywords:** *Corynebacterium glutamicum*, ChIP-seq, AmtR, creatine, creatinine, MFS transporter

## Abstract

In the genus *Corynebacterium*, AmtR is a key component of the nitrogen regulatory system, and it belongs to the TetR family of transcription regulators. There has been much research on AmtR structure, functions, and regulons in the type strain *C. glutamicum* ATCC 13032, but little research in other *C. glutamicum* strains. In this study, chromatin immunoprecipitation and massively parallel DNA sequencing (ChIP-seq) was performed to identify the AmtR regulon in *C. glutamicum* ATCC 14067. Ten peaks were obtained in the *C. glutamicum* ATCC 14067 genome including two new peaks related to three operons (RS_01910-RS_01915, RS_15995, and RS_16000). The interactions between AmtR and the promoter regions of the three operons were confirmed by electrophoretic mobility shift assays (EMSAs). The RS_01910, RS_01915, RS_15995, and RS_16000 are not present in the type strain *C. glutamicum* ATCC 13032. Sequence analysis indicates that RS_01910, RS_01915, RS_15995, and RS_16000, are related to the degradation of creatine and creatinine; RS_01910 may encode a protein related to creatine transport. The genes RS_01910, RS_01915, RS_15995, and RS_16000 were given the names *crn*A, *cre*T, *csh*A, and *hyu*B, respectively. Real-time quantitative PCR (RT-qPCR) analysis and sfGFP (superfolder green fluorescent protein) analysis reveal that AmtR directly and negatively regulates the transcription and expression of *crn*A, *cre*T, *csh*A, and *hyu*B. A growth test shows that *C. glutamicum* ATCC 14067 can use creatine or creatinine as a sole nitrogen source. In comparison, a *cre*T deletion mutant strain is able to grow on creatinine but loses the ability to grow on creatine. This study provides the first genome-wide captures of the dynamics of *in vivo* AmtR binding events and the regulatory network they define. These elements provide more options for synthetic biology by extending the scope of the AmtR regulon.

## Introduction

*Corynebacterium glutamicum* is a Gram-positive and generally regarded as safe (GRAS) microorganism with less endotoxicity. *C. glutamicum* usually adapts to different growth environments and pressures in industrial applications and laboratory research and it can be widely used for the production of amino acids, organic acids, and other products related to health, cosmetics, and food (Becker et al., 2011; Binder et al., 2013). Genome sequencing (Tauch et al., 2002; Kalinowski et al., 2003; Lv et al., 2012), transcriptome sequencing (Hayashi et al., 2002; Fan et al., 2021), metabolome (Krömer Jens et al., 2004; Feith et al., 2020), and proteome (Hermann et al., 2001; Jiang et al., 2020) analyses gradually elucidated the perfected genome-scale metabolic map of *C. glutamicum*, expanding the scope of its application. At present, not only the type-strain *C. glutamicum* ATCC 13032, but many non-type strains have also been studied and applied, for example, *C. glutamicum* ATCC 14067 (Huang et al., 2017; Zhang et al., 2021), *C. glutamicum* R (Kubota et al., 2015), *C. glutamicum* AS 1.542 (Chen et al., 2012), *C. glutamicum* ATCC 13869 (Kikuchi et al., 2006; Liu et al., 2018b). These strains have apparent differences in several phenotypic characteristics (Yang and Yang, 2017).

Gene transcription and expression is regulated by transcriptional factors, including sigma factors and two-component systems that are essential for stabilizing cell homeostasis (López-Maury et al., 2008). The research on transcription factors in *C. glutamicum* was mainly focused on the type strain *C. glutamicum* ATCC 13032. But there are specific regulatory elements in other strains that give those strains different metabolic capabilities. Most *C. glutamicum* strains cannot use l-arabinose as a carbon source, however, the gene cluster for l-arabinose utilization and its regulation have been reported in *C. glutamicum* ATCC 31831 (Kuge et al., 2015). Fifteen *paa* (*paa*TK, *paa*ABCDEGJFH, *paa*I, *paa*Y, and *paa*Z) genes encode the phenylacetic acid degradation pathways that regulated by PaaR in *C. glutamicum* AS 1.542 (Chen et al., 2012). A shikimate transporter, regulated by ShiR in *C. glutamicum* R, was identified and a shikimic acid biosensor was constructed from ShiR for monitoring shikimate synthesis in *C. glutamicum* RES167 (Kubota et al., 2015; Liu et al., 2018a). The regulatory elements in such strains provide more options for synthetic biology.

*C. glutamicum* can use a wide range of nitrogen compounds as nitrogen sources, and AmtR is a critical component of the nitrogen regulatory system that belongs to the TetR family of transcription regulators (Jakoby et al., 2000). In the type strain *C. glutamicum* ATCC 13032, there has been much research on AmtR structure, function, and regulons (Jakoby et al., 2000; Bendt et al., 2004; Hasselt et al., 2011). A total of 35 genes are regulated by AmtR, including the genes that encode the ammonium transporters *amt*A, *amt*B, the urea uptake system *urt*ABCDE, the l-glutamate uptake system *glu*ABCD, the creatinine transporter *crn*T, the ABC-type transport systems Ncgl 1915-1918 involved in the transport of nitrogen sources. The *ure*ABCEFGD, *glt*BD, *gdh*, *gln*A, and *cod*A encoding proteins are involved in nitrogen metabolism (Hasselt et al., 2011). In addition, the *mez* gene encoding the malic enzyme involved in carbon metabolism is also regulated by AmtR (Gourdon et al., 2000). The binding of AmtR to its regulon promoter region relies on conserved sequences and no small molecule effectors have been identified. A PII-type signal transduction protein GlnK, adenylylated at Tyr51, has been proposed to derepress expression of the AmtR regulons (Strösser et al., 2004).

*C. glutamicum* ATCC 14067 is an important industrial workhorse employed as an l-glutamate producer and a parental strain for breeding the producers of l-lysine and l-glutamine (Li et al., 2007; Jianzhong et al., 2014; Lv et al., 2021). In this study, ChIP-seq was performed to identify the regulon of AmtR in the non-type strain *C. glutamicum* ATCC 14067. Four new target genes (RS_01910, RS_01915, RS_15995, and RS_16000) were determined. The proteins encoded by these new target genes may be related to creatine and creatinine degradation, and these genes were given the names crnA, creT, hyuB, and cshA, respectively. Creatine, a component of the dissolve-free amino acid (DFAA) pool, is a metabolite of metazoans. It is found in the excretions of different animals, including earthworms and birds (Wyss and Kaddurah-Daouk, 2000). In addition, some phytoplankton may also produce creatine *via* metabolism (Wawrik et al., 2017). It has been reported that *C. glutamicum* ATCC 13032 cannot utilize creatine as a carbon or nitrogen source. We investigated the abilities of *C. glutamicum* ATCC 14067 to degrade creatine. The result show *C. glutamicum* ATCC 14067 could grow in CGXII medium with creatine as a sole nitrogen source, and CreT is related to creatine transport. Real-time quantitative PCR (RT-qPCR) analysis and sfGFP (superfolder green fluorescent protein) analysis reveal that AmtR directly and negatively regulate the transcription and expression of crnA, creT, hyuB, and cshA.

## Materials and Methods

### Bacterial Strains, Media, and Culture Conditions

The strains and plasmids used in this study are listed in Supplementary Table S1. *Escherichia coli* DH5α was used for gene cloning, and BL21 (DE3) was used for protein expression. *E. coli* strains were grown in Luria-Bertani (LB) (10 g/L tryptone, 5 g/L yeast extract, and 10 g/L sodium chloride) medium at 37°C or 16°C. *C. glutamicum* strains were grown overnight in BHI medium (37 g/L brain-heart infusion) (Becton, Dickinson and Co.) and then cultured in fresh BHI medium or CGXII medium (Keilhauer et al., 1993) at 30°C. Cells grown in the CGXII medium, were centrifuged and washed twice with CGXII and then cultured in fresh CGXII medium. If necessary, CGXII medium was supplemented with 10 mM creatine or creatinine as a nitrogen and carbon source. Strain growth was monitored by measuring the optical density (OD) of the cultures at 600 nm. The medium was supplemented with antibiotics at the following concentrations: kanamycin (Kan): 50 μg/ml for *E. coli* and 25 μg/ml for *C. glutamicum*, chloramphenicol (Chl): 15 μg/ml for *E. coli* and 7.5 μg/ml for *C. glutamicum*.

### Construction of the *amt*R, *cre*T Deletion Mutants and *amt*R-3Flag Complementation in *C. glutamicum* ATCC 14067

Standard DNA cloning, Gibson assembly, and transformation procedures were employed (Sambrook and Russell, 2001; Gibson et al., 2009). The *amt*R and *cre*T deletion mutants (△*amt*R and △*cre*T) in *C. glutamicum* ATCC 14067 were constructed using homologous recombination (Huang et al., 2017). The DNA fragments of the upstream and downstream homology arms of *amt*R and *cre*T were amplified by PCR using the primers *amt*R-L/*amt*R-L-lox71 and *amt*R-R/*amt*R-R-lox66, *cre*T-L/*cre*T-L-lox71 and *cre*T-R/*cre*T-R-lox66 overlapping PCR with a Cre-kan cassette to construct a self-excisable cassette, respectively. The self-excisable cassettes were introduced into *C. glutamicum* ATCC 14067 carrying the recombinase-exonuclease pairs by electroporation (Takano et al., 2008). BHI solid medium supplemented with Kan 25 μg/ml and Chl 7.5 μg/ml was used for recombinant selection. Theophylline (1 mM) was used for inducing Cre expression that mediated intermolecular excision. The DNA fragments for *amt*R and *cre*T deletion strains were confirmed by DNA sequencing (Sangon Biotech, China). A 3Flag tag was added to the C-terminal of AmtR, and the *amt*R-3Flag fragment was amplified using the primers *amt*R-3Flag-S/*amt*R-3Flag-A. A plasmid backbone was amplified from plasmid pEC-XK99E using the primers 99E-S/99E-A. Then the plasmid backbone and the *amt*R-3Flag fragment were assembled by Gibson assembly to construct the plasmid pEC-XK99E-*amt*R-3Flag, which was introduced into the △*amt*R strains by electroporation. The primers used in this study are listed in Supplementary Table S2.

### Western Blot Analysis

Complemented strain *C. glutamicum* 14067-△*amt*R:*amt*R-3Flag was grown in BHI medium, and AmtR-3Flag production was induced using 0.5 mM IPTG (isopropyl -*β*-d-thiogalactopyranoside) for 8 h. AmtR-3Flag production was not induced with 0.5 mM IPTG as the negative control. The cells were harvested by centrifugation and washed with PBS buffer (150 mM NaCl, 3 mM KCl, 10 mM Na_2_PO_4_, 3 mM KH_2_PO_4_, pH 7.5), suspended in PBS buffer to normalize the culture densitiy based on the OD_600_ value, and disintegrated with silica beads (0.1 mm) for 12 cycle of 30 s at a speed rating of 6.0 with 3 min resting intervals by Bead Ruptor 12 (OMNI International, United States). Soluble extracts were fractionated on a 12% denaturing polyacrylamide gel before being transferred to a polyvinylidene difluoride (PVDF) membrane (Millipore, Bedford, MA, United States). The membrane was blocked with 3% bovine serum albumin and incubated overnight at 4°C with a 1:2,000 dilution of Flag-specific (Sigma-Aldrich, St. Louis, MO, United States) mouse antiserum, and incubated with a 1:5,000 dilution of horseradish peroxidase-conjugated goat anti-mouse IgG (Santa Cruz Biotechnology, Dallas, TX, United States). Finally, the immunoreactive protein bands were visualized with an ECL reagent (Thermo Fisher Scientific Inc., Waltham, MA).

### Overexpression and Purification of AmtR

AmtR-His_6_ expression plasmid constructed by PCR using the primers *amt*R-S/*amt*R-A was inserted into pET28a, which had a His_6_-tag at its C-terminus. The plasmid was confirmed by DNA sequencing (Sangon Biotech, China). AmtR with a C-terminal His_6_-tag was expressed in *E. coli* BL21, plasmid carrying cells were grown to an OD_600_ of 0.6 at 37°C in LB medium, and protein production was induced using 0.5 mM IPTG at 16°C for 16 h. Cells were harvested by centrifugation and suspended in buffer A (100 mM Tris-HCl, 100 mM NaCl, pH 7.5), then supplemented with Mini protease inhibitor cocktail tablets (Roche, Germany). Cells disrupted by sonication at 4°C, and cell debris was removed by centrifugation at 4°C (15,000 g for 20 min). The AmtR-His_6_ was purified by 5 ml nickel affinity chromatography using Ni-NTA agarose (Novagen, United States).

### Total RNA Extraction and RT-qPCR Analysis

The *C. glutamicum* ATCC 14067 wild-type and △*amt*R strains were grown overnight in BHI medium, inoculated into fresh CGXII medium to an OD_600_ of 0.2, and cultured for 8 h before the extraction of RNA. If necessary, CGXII medium was supplemented with 10 mM creatine as a nitrogen source. Total RNA was extracted using an RNA extraction kit (Tiangen, Beijing, China) with on-column DNaseI treatment. The final RNA concentrations and purities were determined on a Thermo Scientific NanoDrop spectrophotometer; equal amounts of RNA (1 μg) were used to generate cDNA (Toyobo, Tsuruga, Japan) using 6-mer random primers. Primers for various genes (Supplementary Table S2) were designed using Primer Premier6 software. Three independent RT-qPCR experiments were performed, and each experiment was run in triplicate. The reactions were run on an Applied Biosystems 7500 real-time system (Applied Biosystems), and the transcript levels were normalized to the 16S rRNA level in each sample using the ∆∆C_T_ method.

### Construction of sfGFP Reporter Plasmids and Fluorescence Assay

Promoters of *cre*T, *csh*A, and *hyu*B were amplified from genomic DNA with the primers *cre*T-S/*cre*T-A, *csh*A-S/*csh*A-A, and *hyu*B-S/*hyu*B-A, respectively. The plasmid backbone was amplified from plasmid pEC-XK99E with the primers 99E-sfGFP-S/99E-sfGFP-A. The promoters were then assembled to the plasmid backbone by Gibson assembly to construct the plasmids pEC-XK99E-P*cre*T-sfGFP, pEC-XK99E-P*csh*A-sfGFP, and pEC-XK99E-P*hyu*B-sfGFP. These plasmids were introduced into the *C. glutamicum* ATCC 14067 wild-type and △*amt*R strains by electroporation.

The wild-type *C. glutamicum* ATCC 14067 strain and the △*amt*R strain with reporter plasmids were grown overnight in BHI medium, the cells washed with CGXII medium, then inoculated into fresh CGXII medium to an OD_600_ of 0.2. If necessary, CGXII medium was supplemented with 10 mM creatine as a nitrogen source. After cultivation at 30°C for 8 h, the cells were washed twice with PBS buffer and resuspended in 200 μl PBS buffer, in 96-well plates for measurement of GFP fluorescence. Fluorescence was assessed at an excitation wavelength of 488 nm and an emission wavelength of 520 nm using a multifunctional microplate reader (Infinite M200, Tecan, Switzerland).

### ChIP-Seq

The ChIP-seq protocol was based on previous experiments with *C. glutamicum* (Jungwirth et al., 2013). Strain *C. glutamicum* ATCC-14067-△*amt*R:*amt*R-3Flag was grown at 30°C in BHI medium, and AmtR-3Flag production was induced using 0.5 mM IPTG for 8 h. AmtR-3Flag production was not induced with 0.5 mM IPTG as the negative control. To achieve protein-DNA crosslinking *in vivo*, a final concentration of 1% formaldehyde was added to the cultures, which was incubated at room temperature for 15 min with gentle shaking. A final concentration of 125 mM glycine was added to stop crosslinking. Cells were harvested by centrifugation, washed twice with a complete protease inhibitor cocktail (Roche) in an ice-cold Tris buffer (20 mM Tris-HCl pH 7.5, 150 mM NaCl), and stored at −80°C. To prepare lysates, the pellets were resuspended in FA-1 buffer (HEPES-KOH at 50 mM [pH 7.5], NaCl at 140 mM, EDTA at 1 mM, Triton X-100 at 1%, and complete protease inhibitor cocktail). One ml sample of this cell suspension was mixed with 0.6 g of 0.1 mm silica beads (BioSpec Products, United States), and the cells were disrupted for 12 cycles of 30 s at a speed rating of 6.0 with 3 min resting intervals by Bead Ruptor 12 (OMNI International, United States). The cell debris was removed by centrifugation at 14,000 g for 15 min at 4°C, and the DNA in the supernatant was sheared to an average length of 200–500 bp by sonication in a water bath (Bioruptor, Diagenode). The lysates were pre-cleared with 30 μl of ChIP magnetic A + G beads (Merck Millipore). The rest of the pre-cleared lysates were incubated overnight at 4°C with monoclonal anti-Flag M2 (Sigma-Aldrich). Protein-DNA complexes were immunoprecipitated with 50 μl of ChIP magnetic A + G beads for 4 h at 4°C and subsequently washed sequentially with low-salt washing buffer (0.1% SDS, 1% Triton X-100, 2 mM EDTA, 20 mM Tris-HCl pH 8.1, 150 mM NaCl), with high-salt washing buffer (0.1% SDS, 1% Triton X-100, 2 mM EDTA, 20 mM Tris-HCl pH 8.1, 500 mM NaCl), with LiCl washing buffer (0.25 M LiCl, 1% NP-40, 1% deoxycholate, 1 mM EDTA, 10 mM Tris-HCl pH 8.1) and finally twice with TE buffer (10 mM Tris-HCl pH 8.1, 1 mM EDTA). The magnetic beads were resuspended in 200 μl of elution buffer (ChIP kit, 17-10086, Merck Millipore). Crosslinking was reverted for 8 h at 65°C. Proteinase K and RNase A were used to remove protein and RNA, respectively. The DNA was extracted with phenol-chloroform and was used for ChIP-seq library preparation. The library was constructed by Novogene Corporation (Beijing, China). Subsequently, pair-end sequencing of sample was performed on Illumina platform (Illumina, CA, United States). The ChIP-seq reads were aligned to the *C. glutamicum* ATCC 14067 genome using BWA mem (v 0.7.12). The enriched peaks were then identified using MACS (v 2.1.0) software (Zhang et al., 2008).

### Electrophoretic Mobility Shift Assay

DNA-binding was determined by Electrophoretic Mobility Shift Assay (EMSA). The fragments P*cre*T-1, P*cre*T-2, P*hyu*B-1, P*hyu*B-2, and P*hyu*B-3 covering the putative AmtR binding sequence in *crn*T, *hyu*B, and *csh*A promotor were annealed using two complementary single-stranded oligonucleotides as described previously (Kraxner et al., 2019).

Purified AmtR was mixed with promoter fragments according to the manufacturer’s protocol (LightShift Chemiluminescent EMSA Kit, Thermo); a total volume of 20 μl contained 1 × binding buffer (10 mM Tris, 50 mM KCl, 1 mM DTT, pH 7.0), 5 mM MgCl, 10 mM EDTA, 2.5% glycerol, 0.05% NP-40, 50 ng/μl Poly (dIdC). The mixture was run on 6% Native-PAGE (polyacrylamide gels) at 100 V in 0.5 × TBE (45 mM Tris, 45 mM boric acid, 2 mM EDTA, pH 8.3). The DNA probe was detected using GelRed.

### Bioinformatics Data Analysis

The gene sequence of *C. glutamicum* ATCC 14067 used in this study was obtained from the GenBank file for NZ_CP022614. Databank searches were performed using BLAST (https://blast.ncbi.nlm.nih.gov/Blast.cgi) (Altschul et al., 1990). The putative -35, -10 elements and transcription start site (TSS) in the promoters were predicted using an online website (http://www.fruitfly.org/seq_tools/promoter.html) (Reese, 2001). The sequence analysis tool MEME (http://meme-suite.org/) (Bailey et al., 2015) was used for the identification of AmtR binding motif in the genus *Corynebacterium*.

## Result

### Genome-Wide Identification of the AmtR Regulon by ChIP-Seq

**FIGURE 1:**
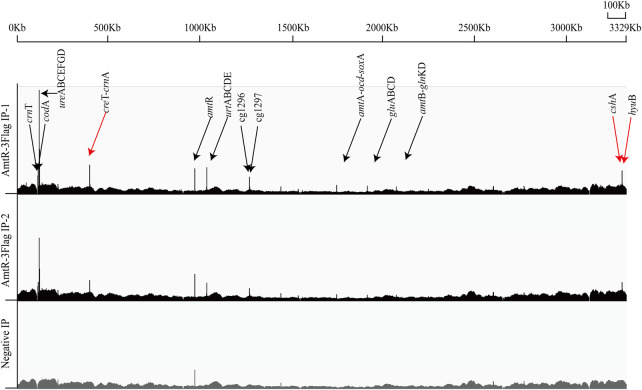
Genome-wide *in vivo* identification of the AmtR binding sites in *C. glutamicum* ATCC 14067. AmtR-3Flag IP reads were plotted against the number of reads from the Negative IP. Black arrows show the verified genes; red arrows show the new target genes. In AmtR-3Flag IP, AmtR-3Flag production was induced using 0.5 mM IPTG for 8 h; AmtR-3Flag production was not induced with 0.5 mM IPTG as the negative control.

AmtR acts as a transcriptional regulator and plays an essential role in the genus *Corynebacterium*. Previous studies have identified the target genes of AmtR by transcriptomics and EMSA (Hasselt et al., 2011; Chen et al., 2013). These studies mainly focused on the type strain *C. glutamicum* ATCC 13032. Due to the different environments and selection pressure during evolution, various strains have apparent differences in several phenotypic characteristics (Yang and Yang, 2017). To identify the direct binding loci of AmtR in *C. glutamicum* ATCC 14067, the pEC-XK99E-*amt*R-3Flag plasmid was used to express the AmtR protein fused with a C-terminal 3Flag-tag and driven by the *lac* promoter in the ∆*amt*R strain. Western blotting confirmed the expression of AmtR-3Flag (Supplementary Figure S1). AmtR-3Flag IP and negative IP DNA were pooled and subjected to Illumina sequencing. To identify the AmtR binding regions, ChIP-seq peaks obtained from the AmtR-3Flag IP samples were compared to the negative IP sample. On ChIP-seq maps, the fold change of peaks above 1.5 was fixed as the minimum cut-off value for AmtR peak calling. On this basis, ten AmtR peaks were detected (Figure 1). Nine of the peaks were located in the promoter regions, and related to 26 verified genes and 4 new target genes (Supplementary Table S3). The other one of the peaks was located in the coding region of argR, and the related sequence did not have a putative AmtR binding site. EMSA was used to detect the direct interactions of AmtR with the DNA fragment (argR-EMSA, 398 bp) that related to the peak located in the coding region of argR. The positive control is the promoter region of *cre*T (PcreT, 444 bp), DNA fragments obtained by PCR amplification. AmtR could not bind and shift the argR-EMSA (Supplementary Figure S2).

### Validation of Novel AmtR Targets

**FIGURE 2:**
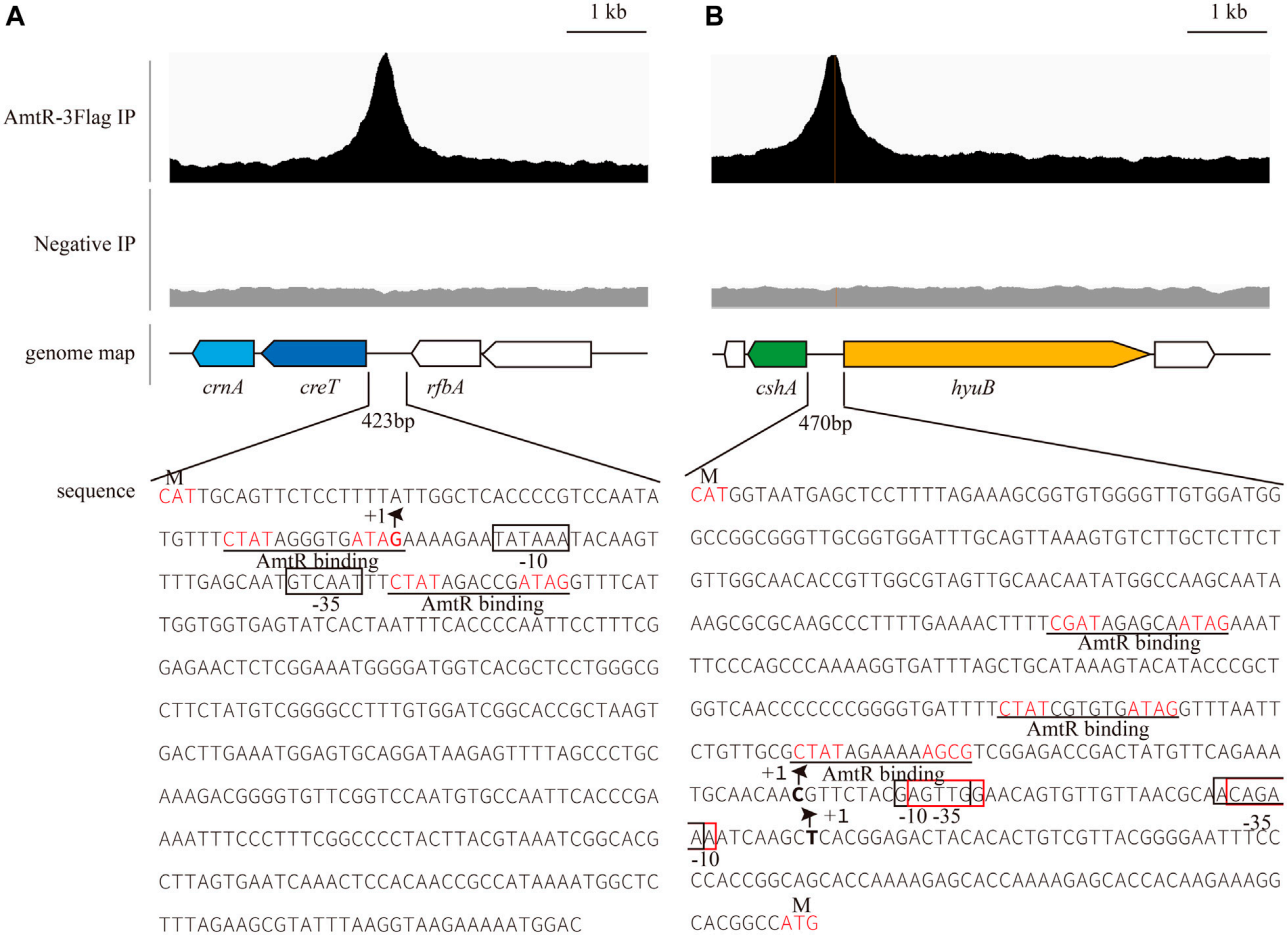
AmtR ChIP-seq peaks of selected genes. The putative palindrome sequence is shown in red; the AmtR binding sequences are predicted by MEME and underlined. +1 indicates the transcription start site (TSS), and putative -35, -10 elements and TSSs in the promoters of *cre*T-*crn*A, *csh*A, and *hyu*B were predicted using the online website (http://www.fruitfly.org/seq_tools/promoter.html) (Reese, 2001). **(A)** Putative -35 and -10 elements in the promoter of *cre*T-*crn*A enclosed by black rectangles. **(B)** Putative -35 and -10 elements in the promoter of *csh*A enclosed by black rectangles, -35 and -10 elements in the promoter of *hyu*B enclosed by red rectangles.

**FIGURE 3:**
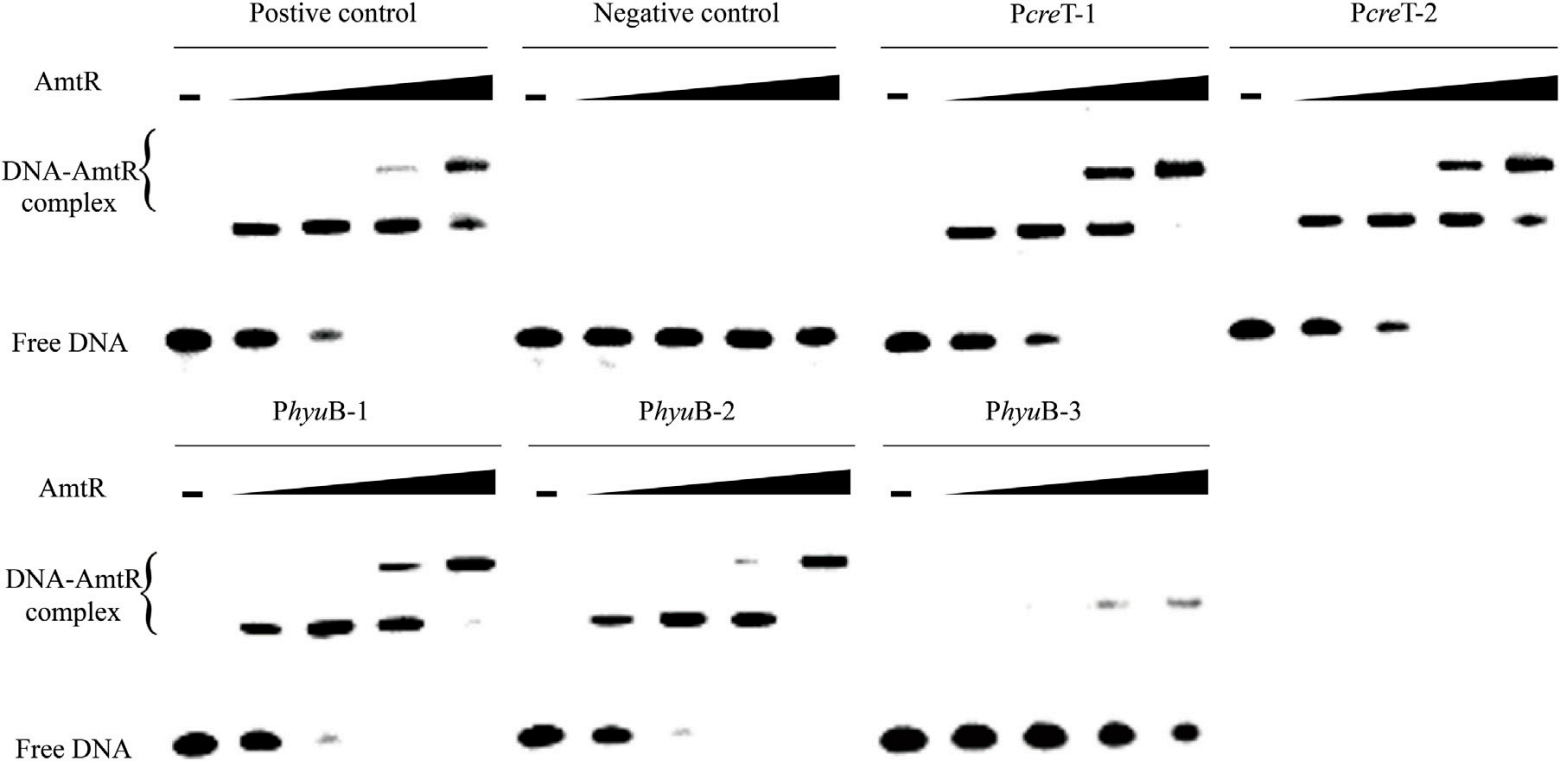
AmtR interacts *in vitro* with different DNA fragments. Minus represents that AmtR was not added, the lower band is the free DNA that is unbound with AmtR. Black triangle represents that AmtR was gradually increasing. The DNA fragments were annealed using two complementary single-stranded oligonucleotides. The positive control is the protomer region of *amt*A reported, and the negative control is the protomer region of *hyu*B that does not contain the AmtR binding sequence. Increasing amounts of AmtR (0, 0.1, 0.2, 0.4, and 0.8 μg) and 100 ng DNA fragments were used.

In *C. glutamicum* ATCC 14067, two new peaks related to three operons were identified using ChIP-seq. (Figure 2). According to a previous prediction by CoryneRegNet 7.0 (Parise et al., 2020), CEY17_RS01915 is the first gene in a two-gene operon encoding putative MFS transporter and cretininase, and CEY17_RS15995 and CEY17_RS16000 are single gene operons encoding putative *N*-carbamoylsarcosine amidase and hydantoin utilization protein HyuB, respectively. The RS_01910, RS_01915, RS_15995, and RS_16000 were given the names *crn*A, *cre*T, *csh*A, and *hyu*B in *C. glutamicum*. The putative TSS (Figure 2) of these genes was predicted using the online website (http://www.fruitfly.org/seq_tools/promoter.html). In *C. glutamicum*, previous studies have reported that AmtR has a 14-bp palindromic binding sequence consisting of two conserved 4-bp sequences forming an inverted repeat separated by a random 6-bp spacer (CTAT-N6-ATAG) (Hasselt et al., 2011). The DNA sequence analysis revealed that the promoter of *cre*T had two potential AmtR binding sites, P*cre*T-1 and P*cre*T-2. The intergenic region of *csh*A and *hyu*B had three potential AmtR binding sites, P*hyu*B-1, P*hyu*B-2, and P*hyu*B-3, respectively. P*hyu*B-2 and P*hyu*B-3 in the promoter of *hyu*B are not strictly conserved. To detect the binding of AmtR and potential AmtR binding site, we assayed the purified AmtR binding to the potential AmtR binding sites *in vitro*. AmtR with a C-terminal His_6_-tag was expressed in *E. coli* BL21 and purified by affinity nickel affinity chromatography as described in the Materials and Methods. DNA fragments contained the binding motif were annealed using two complementary single-stranded oligonucleotides (Supplementary Table S2). AmtR was able to bind and shift the DNA fragments (Figure 3). Increasing amounts of AmtR and 100 ng DNA fragments were used. As the amount of AmtR increased, the binding of AmtR with DNA fragments gradually increased. AmtR is strongly bound with P*cre*T-1, P*cre*T-2, P*hyu*B-1, and P*hyu*B-2, and weakly bound with P*hyu*B-3. The positive control was the protomer region of *amt*A previously reported, and the negative control was the protomer region of *hyu*B that did not contain the AmtR binding sequence (Figure 3).

### Utilization of Creatine as an Alternative Nitrogen Source in *C. glutamicum* ATCC 14067

A reciprocal BLASTP search of CreT, CrnA, CshA, and HyuB was done against the genome database in NCBI to identify homologs. CrnA shares 34% sequence identity with creatininase from *Pseudomonas putida* (Beuth et al., 2003), CshA shares 60% sequence identity with *N*-carbamoylsarcosine amidase from *Arthrobacter* sp. (Roma˜o et al., 1992), and HyuB shares 44% sequence identity with hydantoinase from *Pseudomonas* sp. NS671. Hydantoinase in *Pseudomonas* sp. NS671 is encoded by two distinctly different subunits, HyuA and HyuB (Watabe et al., 1992; Ishikawa et al., 1997). No homologous functional protein for CreT has been reported. The model for the location of the *trans*-membrane helices in CreT was predicted by TMHMM v. 2.0 (http://www.cbs.dtu.dk/services/TMHMM) (Supplementary Figure S3). Hydropathy analysis shows that CreT is an integral membrane protein with 11 putative membrane-spanning domains. Interestingly, CreT has 50% similarities with the MFS transporter encoded by the gene located on upstream of creatinase in *P. putida* NBRC 14164 (PP4_RS10800). Since clustered genes usually encode proteins that physically interact, or different enzymes in the same metabolic pathway (Bork et al., 1998; Dandekar et al., 1998), it is possible that *cre*T encodes a protein related to creatine transport. Creatininase catalyzes both the conversion of creatine and creatinine; *N*-carbamoylsarcosine amidase catalyzes the hydrolysis of *N*-carbamoylsarcosine to sarcosine with the liberation of carbon dioxide and ammonia; hydantoinase catalyzes the hydrolysis of hydantoin to *N*-carbamylamino acid. We hypothesized that CreT, CrnA, CshA, and HyuB were related to creatine and creatinine degradation.

**FIGURE 4:**
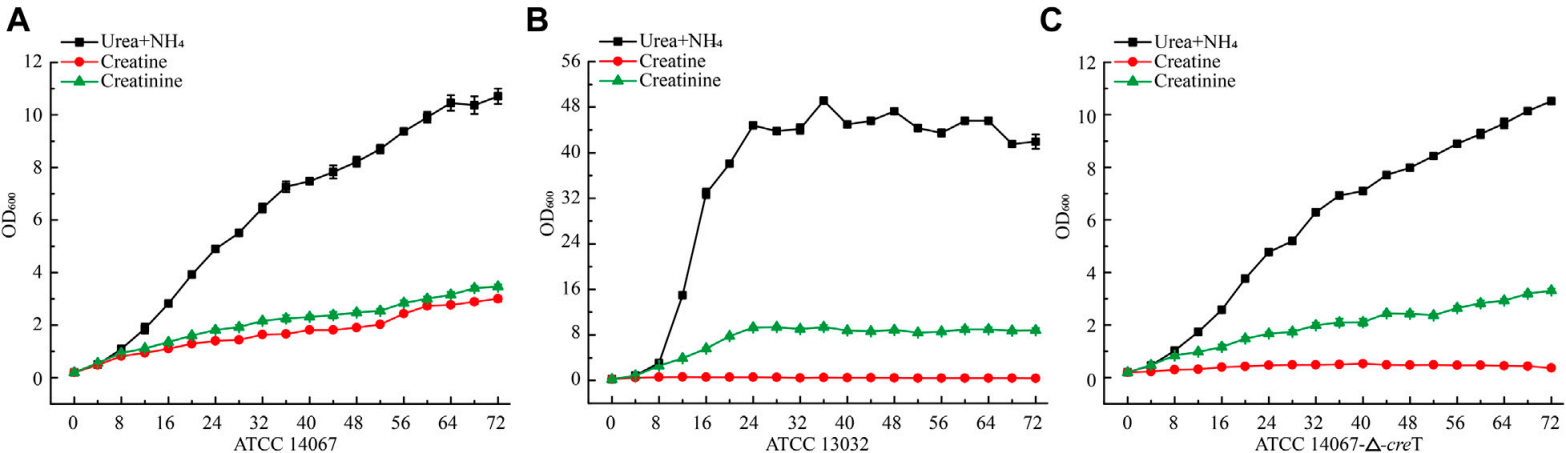
Growth curve of *C. glutamicum* ATCC 14067 wild-type, the △*cre*T, and *C. glutamicum* ATCC 13032 strains with a different nitrogen sources. **(A)** *C. glutamicum* ATCC 14067 grows in CGXII medium with ammonium and urea, 10 mM creatine or 10 mM creatinine as nitrogen sources. **(B)** *C. glutamicum* ATCC 14067-△*cre*T grows in CGXII medium with ammonium and urea, 10 mM creatine or 10 mM creatinine as nitrogen sources. **(C)** *C. glutamicum* ATCC 13032 grows in CGXII medium with ammonium and urea, 10 mM creatine or 10 mM creatinine as nitrogen sources.

**FIGURE 5:**
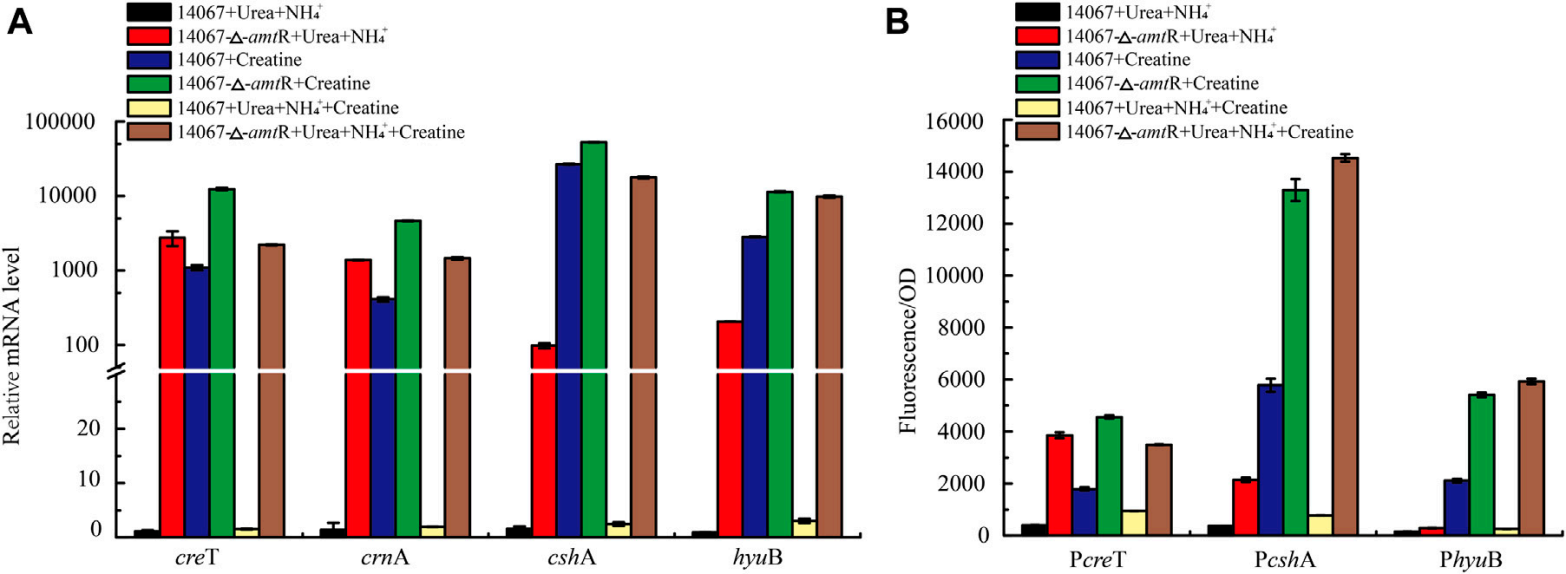
AmtR regulates creatine degradation in *C. glutamicum* ATCC 14067. **(A)** Relative expression of *cre*T, *crn*A, *csh*A, and *hyu*B of *C. glutamicum* ATCC 14067 wild-type and Δ*amt*R strains grown in CGXII medium with creatine or ammonium and urea as nitrogen sources. **(B)** Promoter activity of *cre*T, *csh*A, and *hyu*B of *C. glutamicum* ATCC 14067 wild-type and Δ*amt*R strains grown in CGXII medium with creatine or ammonium and urea as nitrogen sources.

To verify whether *C. glutamicum* ATCC 14067 can degrade creatine and creatinine, we conducted growth assays of *C. glutamicum* ATCC 14067 and *C. glutamicum* ATCC13032 in CGXII medium with creatine or creatinine as a nitrogen source. *C. glutamicum* ATCC 14067 can grow on creatine or creatinine at a concentration of 10 mM (Figure 4A). While C. glutamicum ATCC 13032 is able to utilize creatinine but not creatine as a nitrogen source (Figure 4C), consistent with previous reports. To verify the functions of CreT, CrnA, CshA, and HyuB, the transcripts of *cre*T, *crn*A, *csh*A, and *hyu*B in CGXII medium with creatine or ammonium and urea as nitrogen sources were analyzed by RT-qPCR. In CGXII medium with 10 mM creatine as a sole nitrogen source, the levels of the *cre*T, *crn*A, *csh*A, and *hyu*B transcripts were higher than those growing on ammonium and urea (Figure 5A). To further validate the results above, P*cre*T-sfGFP, P*csh*A-sfGFP, and P*hyu*B-sfGFP reporter plasmids were transformed into the wild-type strain, and promoter activities of P*cre*T, P*csh*A, and P*hyu*B in these strains were observed with 10 mM creatine or ammonium and urea. The GFP fluorescence of P*cre*T, P*csh*A, and P*hyu*B was significantly higher when the strain was grown with creatine than with ammonium and urea (Figure 5B).

In addition, we tested the growth of *C. glutamicum* ATCC 14067 and *C. glutamicum* ATCC 13032 in CGXII medium with 10 mM creatine, 10 mM creatinine, or 10 mM glucose as sole carbon source. CGXII medium without carbon source was used as a negative control. After culturing for 48 h at 30°C, the OD of cultures at 600 nm was measured. *C. glutamicum* ATCC 14067 and *C. glutamicum* ATCC 13032 could not grow in CGXII medium with creatine or creatinine as the carbon source (Supplementary Figure S4).

To further verify the function of CreT in creatine degradation in *C. glutamicum* ATCC 14067, *cre*T was deleted, and the in-frame deletion mutant △*cre*T was obtained. The ∆*cre*T strain could grow as the *C. glutamicum* ATCC 14067 wild-type strain in CGXII medium with ammonium and urea or creatinine as a nitrogen source, but the ∆*cre*T strain lost the ability to grow on creatine. These results demonstrate that *cre*T encodes a protein related to creatine transport in *C. glutamicum* ATCC 14067.

### AmtR Represses Target Gene Transcription and Expression

To investigate the contribution of AmtR to *cre*T, *crn*A, *csh*A, and *hyu*B, the ∆*amt*R strain was constructed using homologous recombination. The *C. glutamicum* ATCC 14067 wild-type and ∆*amt*R strains were cultured in CGXII medium with ammonium and urea or ammonium, urea, and 10 mM creatine as nitrogen sources for 8 h. When ammonium and urea were used as nitrogen sources, compared with the wild-type strain, the *cre*T, *crn*A, *csh*A, and *hyu*B transcript levels in the ∆*amt*R strain were increased significantly. The levels of *cre*T, *crn*A, *csh*A, and *hyu*B increased by 2,427-, 998-, 61-, and 229-fold, respectively (Figure 5A). When the wild-type strain used ammonium, urea, and 10 mM creatine as nitrogen sources, the *cre*T, *crn*A, *csh*A, and *hyu*B transcript levels were higher than those with ammonium and urea as nitrogen sources, but only increased by 1.5-, 1.9-, 2.5-, and 3.0-fold, respectively. The results indicate that creatine could not or very weakly induce the transcription of *cre*T, *crn*A, *csh*A, and *hyu*B when ammonium and urea are abundant. The corresponding sfGFP assays confirmed this result. The P*cre*T-sfGFP, P*csh*A-sfGFP, and P*hyu*B-sfGFP reporter plasmids were transformed into the *C. glutamicum* ATCC 14067 wild-type and △*amt*R strains. The resulting strains were cultured in CGXII medium for 8 h, the sfGFP activities were measured (Figure 5B). Combining RT-qPCR analysis and sfGFP assays, we identified that AmtR represses the transcription and expression of the target genes.

### Proteins Related to Creatine or Creatinine Degradation in the Genus *Corynebacterium* and Related Bacteria

**FIGURE 6:**
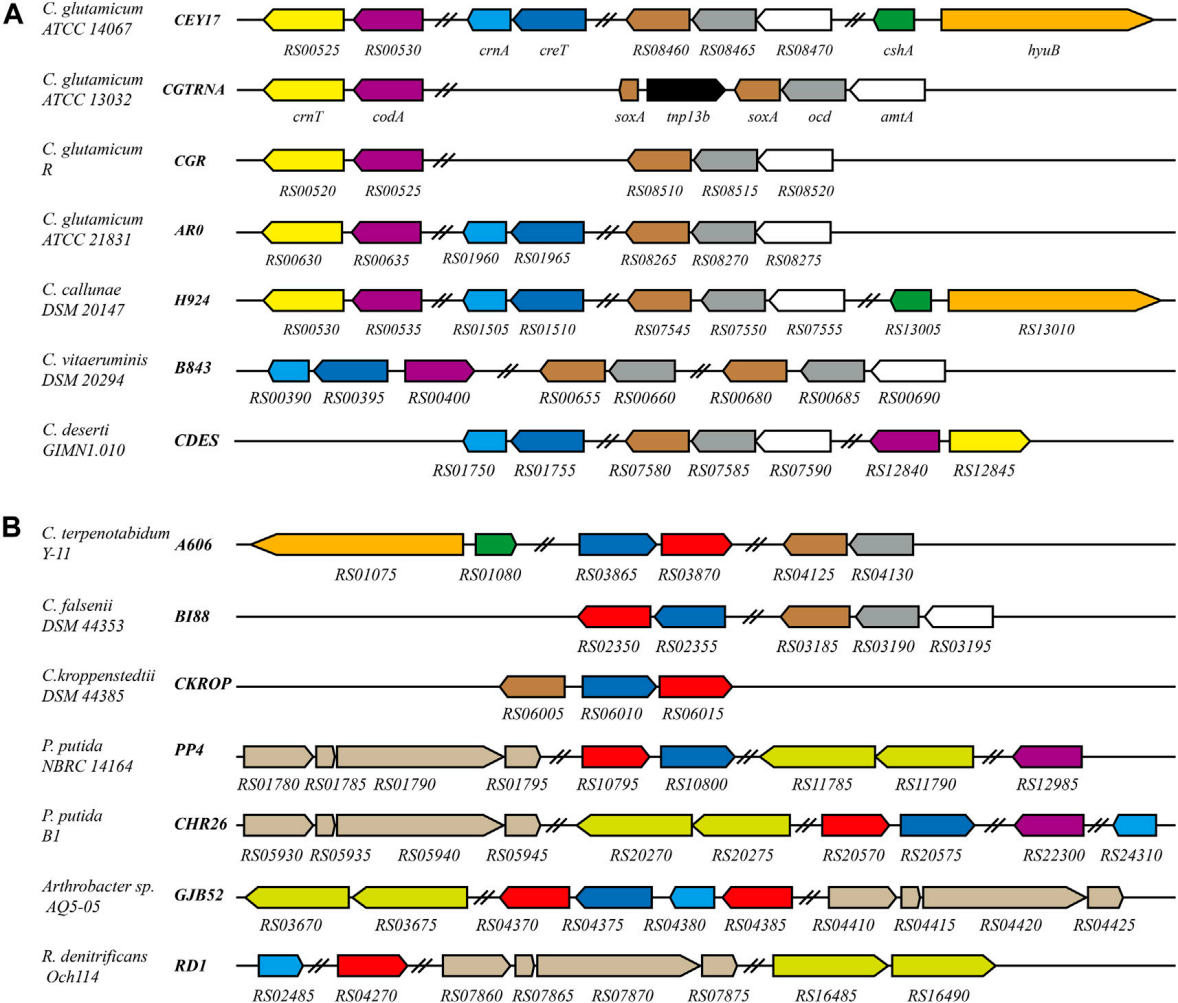
Schematic representation of the creatine and creatinine degradation gene cluster in *C. glutamicum* and related bacteria. Black bold italics indicate locus_tag prefix; homologous genes are presented in the same color. Red represents the gene encoding creatinase (CreA). Navy blue represents the gene encoding the protein related to creatine transport. In *C. glutamicum* ATCC 13032, *sox*A is interrupted by tnp13b. In *P. putida* NBRC 14164, *P. putida* B1, *Arthrobacter* sp. AQ5-05 and *R. denitrificans* Och114, sarcosine oxidase is a heterotetrameric; hydantoinase is a heterodimer. Double slashes indicate that the genes are not continuous. **(A)** The genes related to creatine and creatinine degradation are regulated by AmtR in these strains. **(B)** Homologs of AmtR are not present in these strains.

CrnT and CodA have been reported related to creatinine transport and degradation, and SoxA was predicted as a sarcosine oxidase in *C. glutamicum* ATCC 13032 (Kalinowski et al., 2003). To identify proteins related to creatine or creatinine degradation in bacteria, a reciprocal BLASTP search of CrnT, CodA, CreT, CrnA, SoxA, CshA, and HyuB against the genome database in NCBI was carried out. These proteins were found to be present in some *Corynebacterium* species. In *C. glutamicum*, all proteins related to creatine and creatinine degradation in ATCC 14067 are also present in ATCC 15168, BE, and YI strains. CrnT, CodA, CreT, CrnA, and SoxA are present in 17 strains, including ATCC 21831, ATCC 13869, ZL-1, and so on. CrnT, CodA, and SoxA are present in the other strains. In addition, *Corynebacterium callunae* DSM 20147, *Corynebacterium vitaeruminis* DSM 20294, and *Corynebacterium deserti* GIMN1.010 have different proteins related to creatine and creatinine degradation (Figure 6). Analysis of the promoters’ sequence of genes that encode proteins related to creatine and creatinine degradation revealed that the promoter regions have similar sequences to the AmtR binding site (Supplementary Figure S5).

In *Corynebacterium terpenotabidum* Y-11, *Corynebacterium falsenii* DSM 44353, and *Corynebacterium kroppenstedtii* DSM 44385, A606_RS03870, BI88_RS02350, and CKROP_RS06015 encode a putative creatinase that hydrolyzes creatine to sarcosine and urea. According to the sequence alignment using Clustal Omega, those proteins share 59, 58, and 59% sequence identity with creatinase from *Arthrobacter* sp. TE 1826, respectively (Nishiya et al., 1998). The promoter regions of those genes encoding creatine, and creatinine degradation proteins do not have sequences similar to the AmtR binding site. Further analysis found that these strains do not contain a homolog of AmtR.

There are many bacterial species distinct from *Corynebacterium* that can degrade creatine or creatinine as nitrogen sources. For example, *Pseudomonas* (Yamada et al., 1985), *Arthrobacter* (Nishiya et al., 1998), and *Roseobacter* (Wawrik et al., 2017) strains (Figure 6B). Interestingly, the homologous transporters are ubiquitous and usually adjacent to creatinase or creatininase in these genomes (Figure 6). This supports the speculation that CreT is related to creatine transport. In addition, R. denitrificans Och114 can degrade creatine as a nitrogen source. Still, the genome does not contain CreT homologous protein, which may indicate that creatine can also enter bacterial cells through other pathways.

## Discussion

In bacteria, the PII signal transduction proteins act in conjunction with various transcription factors to control nitrogen metabolism, such as nitrate assimilation through the global nitrogen control factor NtcA in all cyanobacteria characterized to date (Llácer et al., 2010). Nitrogen limitation in enteric bacteria is regulated by NtrBC (Caballero et al., 2005), which activates the expression of over 100 genes; nitrogen metabolism in *Bacillus subtilis* under conditions of nitrogen limitation is regulated by TnrA (Wray et al., 1996); the GlnR controls nitrogen metabolism in *Streptomyces coelicolor* and regulates at least 50 nitrogen response genes (Tiffert et al., 2008). The AmtR is the central nitrogen regulator in *C. glutamicum*, and it has been reported that AmtR in conjunction with the PII signal transduction protein GlnK triggers the dissociation of AmtR from DNA. The conservative sequence of the AmtR binding site contains two conserved 4-bp sequences forming an inverted repeat separated by a random 6-bp spacer, which is CTAT-N_6_-ATAG. In addition, in *Streptomyces avermitilis*, *Rhodococcus jostii* RHA1, *Arthrobacter aurescens*, *Mycobacterium smegmatis*, and *Nocardia farcinica*, both *gln*R and *amt*R-homologous genes are present in the genome. The GlnR is the central nitrogen regulator in those strains, and AmtR has a 22 bp consensus TAtCTGTCa-n2-cGACAGATAT sequence (Chen et al., 2013), similar to the AmtR binding site sequence in *C. glutamicum*. In different species, AmtR and its homologous protein have similar binding sequences.

The binding site of AmtR in the *C. glutamicum* ATCC 14067 genome was identified by ChIP-seq in BHI medium. Ten peaks were obtained in the *C. glutamicum* ATCC 14067 genome including two new peaks related to three operons. ChIP-seq detected a total of 30 genes include four new target genes, fewer than the 35 genes previously reported (Hasselt et al., 2011). There may be some genes not strictly regulated by AmtR. In a nitrogen-rich medium, the transcription and expression of *gdh* and *gln*, which encode l-glutamate dehydrogenase and l-glutamine synthetase, are only loosely controlled by AmtR (Hasselt et al., 2011).

To identify the transcription and expression of creT, *crn*A, cshA, and hyuB regulated by AmtR, we used 50 bp double-stranded DNA containing the potential AmtR binding sites annealed using two complementary single-stranded oligonucleotides. EMSA showed that PcreT-1, PcreT-2, PhyuB-1, and PhyuB-2 have a robust affinity when binding with AmtR, and PhyuB-3 has a very weak affinity with AmtR. PhyuB-1, PhyuB-2, PhyuB-3 are located in the intergenic region of cshA and *hyu*B, cshA is transcribed divergently from *hyu*B. RT-qPCR and other assays were employed to investigate the effect of the △*amt*R strain on the promoter activity of cshA and *hyu*B. The result of the analyses showed that the transcription and expression of cshA and *hyu*B in the ∆*amt*R strain were significantly higher than in the wild-type strain. In previous studies, urea acted as an alternative nitrogen source that could diffuse across the cytoplasmic membrane present in high concentrations in the medium; the transporter of urea is strictly and fully repressed by AmtR in *C. glutamicum* (Siewe et al., 1998). Creatinine acts as a membrane-impermeable nitrogen source, and the genes that code transport and degrade creatinine are limited to nitrogen starvation and strictly regulated by AmtR (Bendt et al., 2004). The ∆*cre*T strain cannot use creatine as a nitrogen source, which indicates that creatine cannot diffuse across the cytoplasmic membrane. The operons *cre*T-*crn*A, *csh*A, and *hyu*B may be limited to nitrogen starvation and be strictly regulated by AmtR.

The new regulons of AmtR in ATCC 14067 are not present in the type strain *C. glutamicum* ATCC 13032. The sequences of CreT, CrnA, CshA, and HyuB were analyzed in *C. glutamicum* strains. CreT and CrnA are present in 17 strains. CreT is related to creatine transport, and its homologous protein is ubiquitous in species that can utilize creatine. The marine bacteria *Roseobacter denitrificans* Och114 can grow on creatine as a nitrogen source, but it has no protein homologous to CreT (Figure 6). However, there may be other ways to take up creatine into the cell. CrnT and CodA related to creatinine transport and degradation are present in all *C. glutamicum* strains. All strains of *C. glutamicum* can use creatinine as a nitrogen source, and only some strains can use creatine as a nitrogen source. It has been reported that creatinine inhibits bacterial replication. *C. glutamicum* can resist creatinine stress in the environment by degrading creatinine to *N*-methylhydantoin and ammonia. Ammonia is used in other pathways, and *N*-methylhydantoin is a dead-end product accumulating in the cells (Bendt et al., 2004). Incomplete degradation of creatinine is detrimental, leading to the arrest of *C. glutamicum* growth when the concentration of creatinine is high. In *C. glutamicum* ATCC 14067, CshA and HyuB can further react with the *N*-methylhydantoin to yield sarcosine, and CrnA can catalyze the conversion of both creatine and creatinine (Beuth et al., 2003). When the creatinine concentration is high, it is converted into creatine that does not inhibit bacteria. If *N*-methylhydantoin is further degraded to sarcosine or creatinine is converted to creatine, it can improve the survival ability of *C. glutamicum* ATCC 14067 when the concentration of creatinine is high. Intraspecies variation of *C. glutamicum* is characterized by different strains with different abilities to degrade creatine and creatinine. Similar characteristics have been seen in *Pseudomonas*. *P. putida* NBRC 14164 has the genes related to the degradation of creatine, but these are not present in *P. putida* KT2440 (Nelson et al., 2002; Ohji et al., 2014). Possibly, these strains gain the ability to degrade creatine via horizontal gene transfer.

*C. glutamicum* ATCC 14067 cannot use creatine or creatinine as the sole carbon source (Supplementary Figure S4). Creatine and creatinine produce ammonia and glycine during the degradation process, but glycine cannot be re-utilized. The glycine cleavage system that degrades glycine is also lacking in *C. glutamicum* (Hüser Andrea et al., 2005), and thus glycine will accumulate in the cells. In some bacteria, creatine or creatinine can be used as the sole carbon source since creatine or creatinine can be degraded into glycine. Serine hydroxymethyltransferase and 10-formyltetrahydrofolate hydrolase further catalyze glycine into serine and pyruvate (Chlumsky et al., 1995; Meskys et al., 2001; Willsey and Wargo, 2015).

**FIGURE 7:**
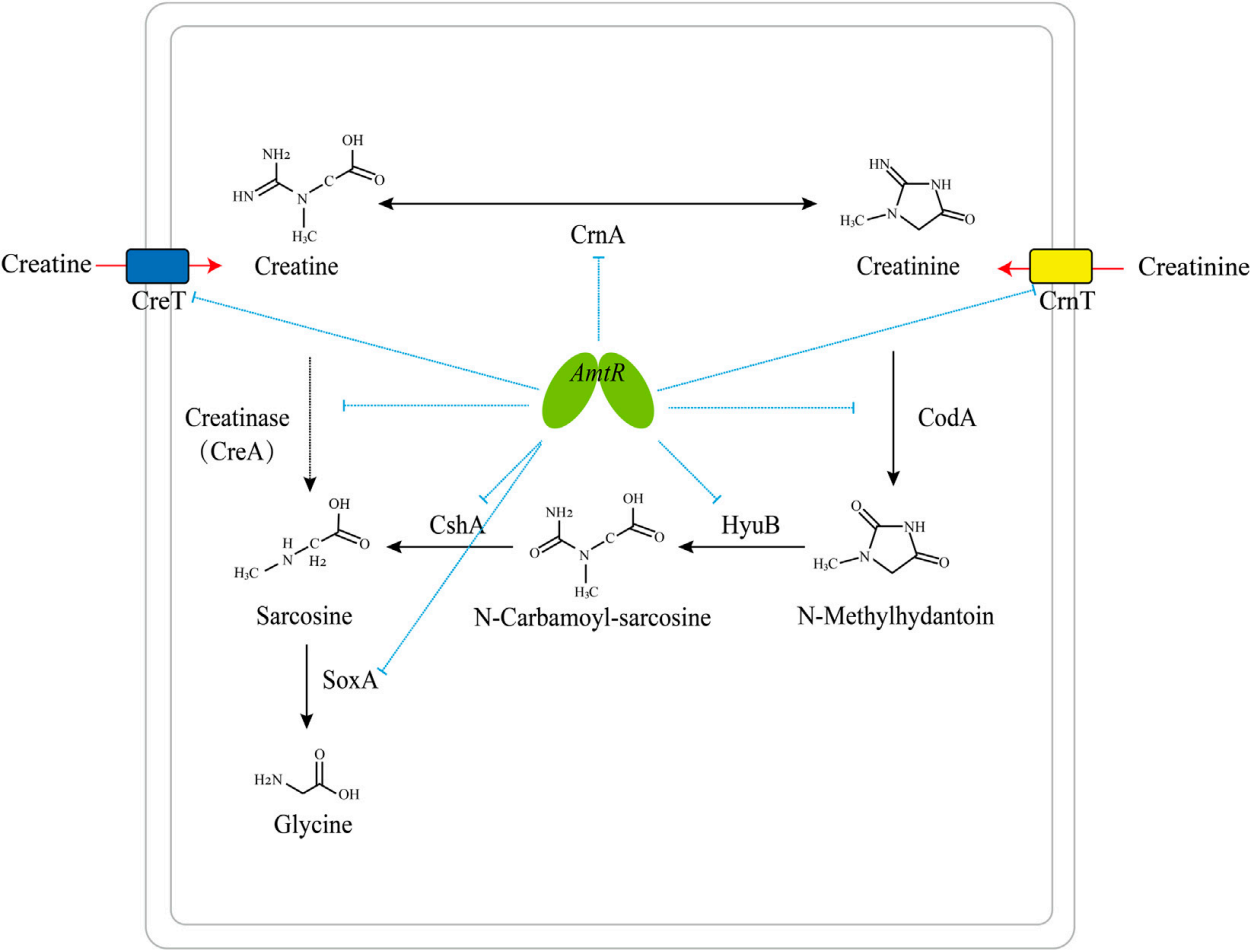
Model of the AmtR regulating creatine and creatinine degradation in *C. glutamicum* ATCC 14067. Blue dotted line: transcriptional repression by AmtR; black arrows: creatine and creatinine degradation pathway; red arrows: the transport of creatine and creatinine; dotted arrow: creatinase (CreA) is not present in *C. glutamicum* ATCC 14067.

It has been reported that the degradation pathway of creatine and related metabolites in bacteria is regulated by transcription factors. Creatinine transport and degradation are regulated by AmtR in *C. glutamicum* (Bendt et al., 2004), and sarcosine catabolism is transcriptionally regulated by GbdR and SouR in *Pseudomonas aeruginosa* (Willsey and Wargo, 2015). The pathway degrading creatine and creatinine into glycine is regulated by AmtR in *C. glutamicum* ATCC 14067 (Figure 7).

## Data Availability

The data presented in the study are deposited in the National Center for Biotechnology Information repository, accession number PRJNA798895.
