# Supplementary material for: Transcriptional Regulation of the Creatine Utilization Genes of Corynebacterium glutamicum ATCC 14067 by AmtR, a Central Nitrogen Regulator
Source: Front Bioeng Biotechnol. 2022 Feb 9;10:816628. doi: 10.3389/fbioe.2022.816628 (PMC8864220; doi:10.3389/fbioe.2022.816628)
Supplement: Supplementary file 1 [file DataSheet1.docx]

**Supplemental material:**

1. **Figures**


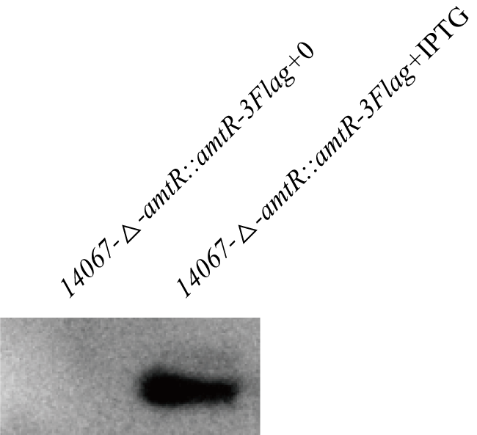


Figure S1. Western blot identification of the expression of AmtR-3Flag. *C. glutamicum* 14067-△*amt*R::*amt*R-3Flag was induced using 0.5 mM IPTG for 8 h. AmtR-3Flag production was not induced with 0.5 mM IPTG as the negative control.


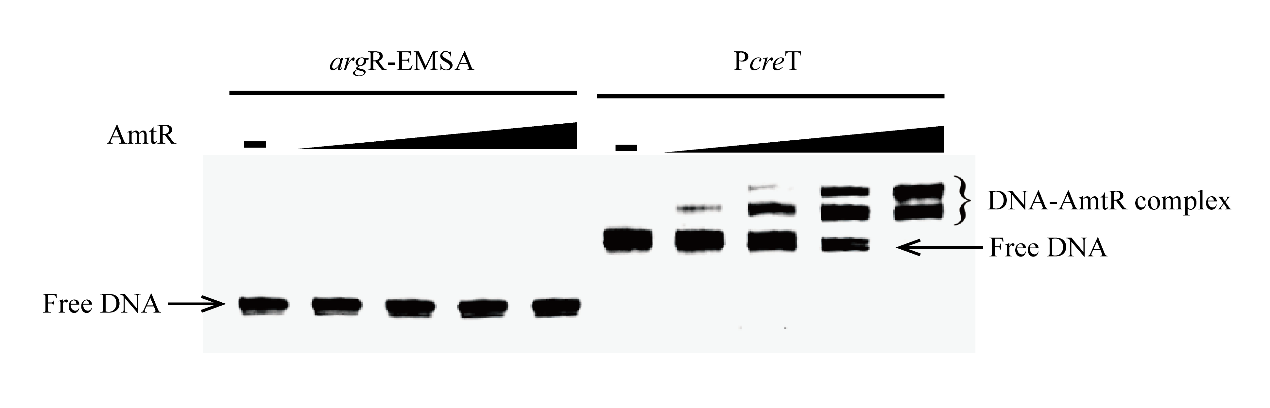


Figure S2. AmtR interacts *in vitro* with different DNA fragments. Minus represents that AmtR was not added, the lower band is the free DNA that is unbound with AmtR. Black triangle represents that AmtR was gradually increasing. DNA fragments obtained by PCR amplification. The positive control is the protomer region of *cre*A. Increasing amounts of AmtR (0 μg, 0.1 μg, 0.2 μg, 0.4 μg, and 0.8 μg) and 100 ng DNA fragments were used.


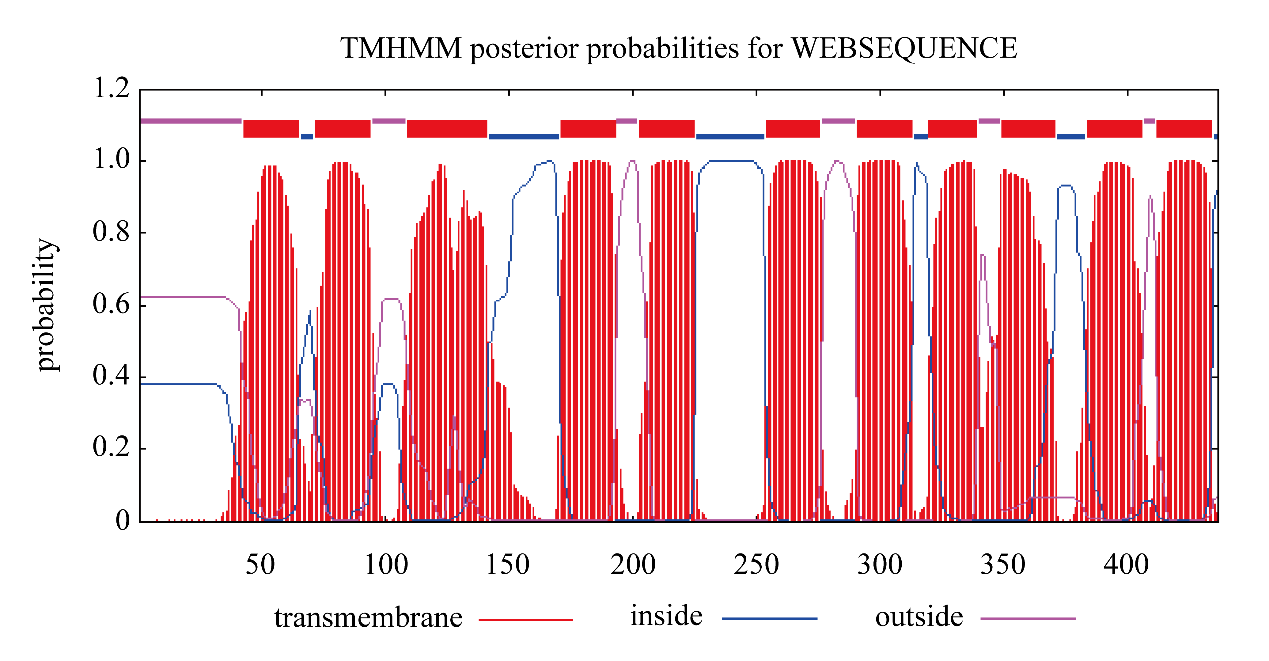


Figure S3. Results of transmembrane helices prediction by TMHMM v. 2.0. Red bars indicate transmembrane domains, blue lines indicate intracellular loops, and magenta lines indicate extracellular loops.


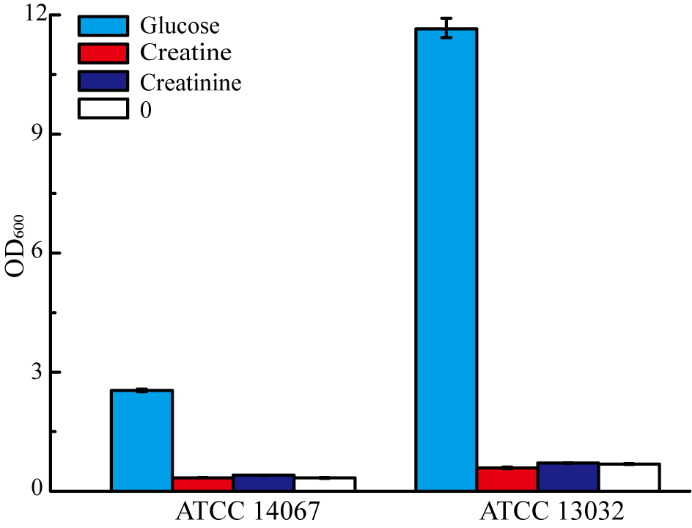


Figure S4. Growth of *C. glutamicum* ATCC 14067 and *C. glutamicum* ATCC 13032 in CGXII medium with glucose, creatine, or creatinine as carbon source. Glucose, creatine, or creatinine show the CGXII medium with 10 mM glucose, 10 mM creatine, or 10mM creatinine as carbon source; 0 indicates the CGXII medium without carbon source as a control. After culturing for 48 h at 30 ℃, the OD of cultures at 600 nm was measured.


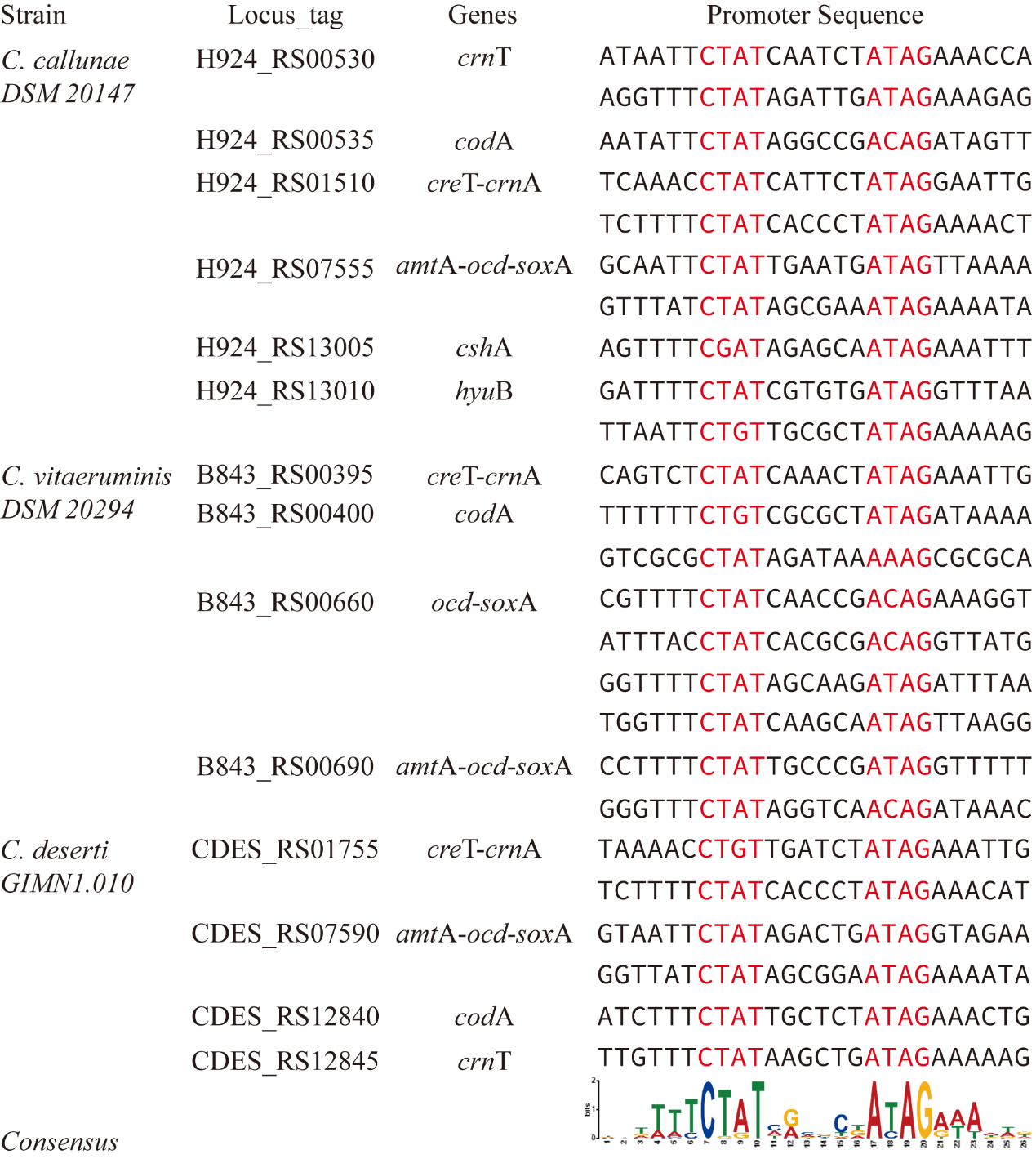


Figure S5. The putative AmtR binding motif. The putative AmtR binding consensus motif was calculated using the putative AmtR binding sequences in the promoters of the genes related to degradation creatine or creatinine in *C. callunae* DSM 20147, *C. vitaeruminis* DSM 20294, and *C. deserti* GIMN1.010. The height of letters represents the frequency of the corresponding nucleotides in the AmtR box.

1. **Tables**

Table S1. Bacterial strains and plasmids used in this study.

| Strains | Characteristics | Origin |
| --- | --- | --- |
| *E. coli* DH5α | *supE44, hsdR17, recA1, thi-1, endA1, lacZ, gyrA96, relA1* | Invitrogen |
| BL21 (DE3) | Carrying a T7 RNA polymerase gene under the control o*f lacUV5* promoter | Invitrogen |
| ATCC 13032 | wild type | ATCC |
| ATCC 14067 | wild type | ATCC |
| ATCC 14067-△*amt*R | *amt*R mutant | this study |
| ATCC 14067-△*cre*T | *cre*T mutant | this study |
| ATCC 14067 △*amt*R::*amt*R-3Flag | amtR mutant with a plasmid pEC-XK99E-*amt*R-3Flag | this study |
| ATCC 14067-P*cre*T-sfGFP | ATCC 14067 with a plasmid pEC-XK99E-P*cre*T-sfGFP | this study |
| ATCC 14067-P*csh*A-sfGFP | ATCC 14067 with a plasmid pEC-XK99E-P*csh*A-sfGFP | this study |
| ATCC 14067-P*hyu*B-sfGFP | ATCC 14067 with a plasmid pEC-XK99E-P*hyu*B-sfGFP | this study |
| ATCC 14067-△*amt*R::P*cre*T-sfGFP | *amt*R mutant with a plasmid pEC-XK99E-P*cre*T-sfGFP | this study |
| ATCC 14067-△*amt*R::P*csh*A-sfGFP | *amt*R mutant with a plasmid pEC-XK99E-P*csh*A-sfGFP | this study |
| ATCC 14067-△*amt*R::P*hyu*B-sfGFP | *amt*R mutant with a plasmid pEC-XK99E-P*hyu*B-sfGFP | this study |
| Plasmids |  |  |
| pEC-XK99E | *C. glutamicum*/*E. coli* shuttle expression vector, Kan^R^ | (Kirchner and Tauch, 2003) |
| pEC-XK99E-*amt*R-3Flag | AmtR-3Flag protein expression in *C. glutamicum* | this study |
| pEC-XK99E-P*cre*T-sfGFP | P*cre*T activily reporter plasmid | this study |
| pEC-XK99E-P*csh*A-sfGFP | P*csh*A activily reporter plasmid | this study |
| pEC-XK99E-P*hyu*B-sfGFP | P*hyu*B activily reporter plasmid | this study |
| pET28a-*amt*R-His6 | AmtR-His6 protein expression in BL21(DE3) | this study |

# Table S2.The primers and oligonucleotides used in this study.

| Designation | Sequence (5'→3') |
| --- | --- |
| *amt*R-L | GATGCCCACGTGGTGCTCACCGG |
| *amt*R-L-lox71 | TGCAGTATAACTTCGTATAATGTATGCTATACGAACGGTATCGCGAGGATTCTTGCCTGCCC |
| *amt*R-R | CATTAGGGAATCGGGGTTGGATCC |
| *amt*R-R-lox66 | ACCCATATAACTTCGTATAGCATACATTATACGAACGGTATCGCAACGACGGCAAGATTCCAAGCCCG |
| *cre*T-L | CTCCGTTAACGATGCCTACCTCCAG |
| *cre*T-L-lox71 | TGCAGTATAACTTCGTATAATGTATGCTATACGAACGGTATACTGCATTCTTAAGGTCTTTTTTG |
| *cre*T-R | ATAAGCACAGATTCCGGCAATGGCG |
| *cre*T-R-lox66 | ACCCATATAACTTCGTATAGCATACATTATACGAACGGTACCTGGATGATCGGTGCCAGTGGC |
| *amt*R-3Flag-S | CACACAGGAAACAGACCATGATGGCAGGAGCAGTGGGA |
| *amt*R-3Flag-A | CGATGTCATGATCTTTATAATCACCGTCATGGTCTTTGTAGTCTTTCGCGTCAGCCTGCTTGATT |
| 99E-S | GGCTGTTTTGGCGGATGAGAGAAGA |
| 99E-A | TTATAAAGATCATGACATCGACTACAAGGATGACGATGACAAGTAAGGCTGTTTTGGCGGATGAG |
| 99E-sfGFP-S | ATGAGCAAAGGAGAAGAACTTTTCAC |
| 99E-sfGFP-A | AACGTAAATGCATGCCGCTTCGC |
| *cre*T-S | CGGCATGCATTTACGTTCTGTTCTTAGTCAGCGCCTTTTGAC |
| *cre*T-A | GTTCTTCTCCTTTGCTCATTGCAGTTCTCCTTTTATTGGCTCACC |
| *csh*A-S | CGGCATGCATTTACGTTGGCCGTGCCTTTCTTGTGGTGC |
| *csh*A-A | GTTCTTCTCCTTTGCTCATGGTAATGAGCTCCTTTTAGAAAGCG |
| *hyu*B-S | CGGCATGCATTTACGTTGGTAATGAGCTCCTTTTAGAAA |
| *hyu*B-A | GTTCTTCTCCTTTGCTCATGGCCGTGCCTTTCTTGTGGTGCT |
| P*crn*T-1-S | ACCACCAATGAAACCTATCGGTCTATAGAAATTGACATTGCT |
| P*crn*T-1-A | AGCAATGTCAATTTCTATAGACCGATAGGTTTCATTGGTGGT |
| P*crn*T-2-S | ATTTATATTCTTTTCTATCACCCTATAGAAACATATTGGACG |
| P*crn*T-2-A | CGTCCAATATGTTTCTATAGGGTGATAGAAAAGAATATAAAT |
| P*hyu*B-1-S | TTGAAAACTTTTCGATAGAGCAATAGAAATTTCCCAGCCCAA |
| P*hyu*B-1-A | TTGGGCTGGGAAATTTCTATTGCTCTATCGAAAAGTTTTCAA |
| P*hyu*B-2-S | CCCGGGGTGATTTTCTATCGTGTGATAGGTTTAATTCTGTTG |
| P*hyu*B-2-A | CAACAGAATTAAACCTATCACACGATAGAAAATCACCCCGGG |
| P*hyu*B-3-S | GTTGCGCTATAGAAAAAGCGTCGGAGACCGACTATGTTCAGA |
| P*hyu*B-3-A | TCTGAACATAGTCGGTCTCCGACGCTTTTTCTATAGCGCAAC |
| EMSA-NC-S | TTATGGCGGTTGTGGAGTTTGATTCACTAAGCGTGCCGATTT |
| EMSA-NC-A | AAATCGGCACGCTTAGTGAATCAAACTCCACAACCGCCATAA |
| EMSA-PC-S | GATTTTGCAAGTTTCTATAGATTGATAGAAAAAGGAGTTTAG |
| EMSA-PC-A | CTAAACTCCTTTTTCTATCAATCTATAGAAACTTGCAAAATC |
| *amt*R-his-S | AACTTTAAGAAGGAGATATACCATGGCAGGAGCAGTGGGAC |
| *amt*R-his-A | TCGAGTGCGGCCGCTTAGTGATGATGATGATGATGTTTCGCGTCAGCCTGCT |
| pET-28a-S | GCGGCCGCACTCGAGCACCACCAC |
| pET-28a-A | CATGGTATATCTCCTTCTTAAAGTT |
| RT-*cre*T-S | ACCGATGGCATTGAGGATGG |
| RT-*cre*T-A | GCAGACGAAGACTCCGCTAA |
| RT-*hyu*B-S | GAACAGATAGTCGCACGCATT |
| RT-*hyu*B-A | AGGTAACGGCTAACGGAAGG |
| RT-*csh*A-S | TTCGGAGCCTACAGCCAAG |
| RT-*csh*A-A | TCTACACCACCACTGCTTATGA |
| RT-*cre*A-S | GCAAGGAGTTCGTCTAGTAGGT |
| RT-*cre*A-A | GTGCTTCCAACGCTCAATTATG |
| RT-16S-S | ACCCTTGTCTTATGTTGCCAG |
| RT-16S-A | TGTACCGACCATTGTAGCATG |
| *arg*R-EMSA-S | GACTCGCACTGCACGCCAAGCTCTC |
| *arg*R-EMSA-A | CGGTGTCATCGCCAGCGATGGTGCC |
| P*cre*T-S | GACATATGGACTTCCACCTCGGGAGTCC |
| P*cre*T-A | TGCAGTTCTCCTTTTATTGGCTCACCCCG |

Table S3. Location of putative AmtR binding sites in AmtR-3Flag IP-1

| Peak | IP 1-Locus | IP 1-Locus | IP 1-Fold_enrichment | IP 2-Fold_enrichment | Genes | Peak class |
| --- | --- | --- | --- | --- | --- | --- |
| Peak1 | 120067-122913 | 120282 - 122624 | 6.84382 | 3.34201 | *ure*ABCEFGD | promoter |
| Peak2 | 1034091-1035933 | 1034186-1036051 | 6.76033 | 3.48429 | *urt*ABCDEF | promoter |
| Peak3 | 112929-116419 | 112290 - 115878 | 5.44995 | 3.29473 | *crn*T, *cod*A | promoter |
| Peak4 | 396035-398943 | 395942 - 397368 | 4.52516 | 2.61973 | *cre*T-*crn*A | promoter |
| Peak5 | 1269463-1270794 | 1269521 - 1270779 | 4.05711 | 2.2686 | cg1296, cg1297 | promoter |
| Peak6 | 3303417-3304952 | 3303656 - 3304768 | 3.45657 | 2.11399 | *csh*A, *hyu*B | promoter |
| Peak7 | 1912090-1912786 | 1912262-1912640 | 2.33721 | 1.53933 | *glu*ABCD | promoter |
| Peak8 | 2070592-2071464 | 2070926 - 2071351 | 2.31208 | 1.58093 | *amt*B-*gln*KD | promoter |
| Peak9 | 1745194-1745714 | 1745299-1745639 | 2.21156 | 1.61213 | *amt*A-*ocd*-*sox*A | promoter |
| Peak10 | 1535737-1536122 | 1535764 - 1536079 | 1.96024 | 1.63293 | *arg*R | coding region |

**References**

Kirchner, O., and Tauch, A. (2003). Tools for genetic engineering in the amino acid-producing bacterium *Corynebacterium glutamicum*. *Journal of Biotechnology* 104(1)**,** 287-299. doi: <https://doi.org/10.1016/S0168-1656(03)00148-2>.
